# Gelsolin Induces Colorectal Tumor Cell Invasion via Modulation of the Urokinase-Type Plasminogen Activator Cascade

**DOI:** 10.1371/journal.pone.0043594

**Published:** 2012-08-21

**Authors:** Jingli Zhuo, Ee Hong Tan, Benedict Yan, Lalchhandami Tochhawng, Manikandan Jayapal, Shiuan Koh, Hwee Kee Tay, Sutherland K. Maciver, Shing Chuan Hooi, Manuel Salto-Tellez, Alan Prem Kumar, Yaw Chong Goh, Yaw Chyn Lim, Celestial T. Yap

**Affiliations:** 1 Department of Physiology, Yong Loo Lin School of Medicine, National University of Singapore, Singapore, Singapore; 2 Beatson Institute for Cancer Research, Glasgow, United Kingdom; 3 Department of Pathology, National University Hospital, Singapore, Singapore; 4 Center of Excellence in Genomic Medicine Research (CEGMR), King Abdulaziz University, Jeddah, Saudi Arabia; 5 Institute of Infection, Immunity and Inflammation, University of Glasgow, Glasgow, United Kingdom; 6 Department of Biomedical Sciences, University of Edinburgh, Edinburgh, United Kingdom; 7 Cancer Science Institute of Singapore, National University of Singapore, Singapore, Singapore; 8 Centre for Cancer Research and Cell Biology, Queen’s University Belfast, Belfast, United Kingdom; 9 Department of Pharmacology, Yong Loo Lin School of Medicine, National University of Singapore, Singapore, Singapore; 10 School of Biomedical Sciences, Faculty of Health Sciences, Curtin University, Bentley, Western Australia, Australia; 11 Department of Biological Sciences, University of North Texas, Denton, Texas, United States of America; 12 Department of Surgery, Singapore General Hospital, Singapore, Singapore; University of Bari & Consorzio Mario Negri Sud, Italy

## Abstract

Gelsolin is a cytoskeletal protein which participates in actin filament dynamics and promotes cell motility and plasticity. Although initially regarded as a tumor suppressor, gelsolin expression in certain tumors correlates with poor prognosis and therapy-resistance. *In vitro*, gelsolin has anti-apoptotic and pro-migratory functions and is critical for invasion of some types of tumor cells. We found that gelsolin was highly expressed at tumor borders infiltrating into adjacent liver tissues, as examined by immunohistochemistry. Although gelsolin contributes to lamellipodia formation in migrating cells, the mechanisms by which it induces tumor invasion are unclear. Gelsolin’s influence on the invasive activity of colorectal cancer cells was investigated using overexpression and small interfering RNA knockdown. We show that gelsolin is required for invasion of colorectal cancer cells through matrigel. Microarray analysis and quantitative PCR indicate that gelsolin overexpression induces the upregulation of invasion-promoting genes in colorectal cancer cells, including the matrix-degrading urokinase-type plasminogen activator (uPA). Conversely, gelsolin knockdown reduces uPA levels, as well as uPA secretion. The enhanced invasiveness of gelsolin-overexpressing cells was attenuated by treatment with function-blocking antibodies to either uPA or its receptor uPAR, indicating that uPA/uPAR activity is crucial for gelsolin-dependent invasion. In summary, our data reveals novel functions of gelsolin in colorectal tumor cell invasion through its modulation of the uPA/uPAR cascade, with potentially important roles in colorectal tumor dissemination to metastatic sites.

## Introduction

Colorectal cancer (CRC) accounts for one of the highest mortality rates from cancer worldwide. The survival rate is highest at about 90% when diagnosed at early stages where tumor growth is localized to primary sites and about 35%–70% in invasive but regional disease. However the occurrence of distant metastasis to the liver or lungs in CRC is a major contributing factor to death, with five-year survival rate at less than 15% [1]. The pathogenesis of CRC from normal colonic epithelium to adenoma is fairly well-characterized and often involves a number of genetic alterations, including mutational activation of oncogenes such as K-ras as well as mutational inactivation of tumor suppressors such as p53 [2] and adenomatous polyposis coli (APC) gene [3]. In contrast, less is known about the molecular mechanisms which convert a non-invasive colorectal neoplasm to one with an invasive phenotype. In most solid tumors, the spread of tumor cells is facilitated by events which result in the detachment of malignant cells from the primary site and subsequent dissemination through tissues and vasculature [4]. This metastatic cascade is critically dependent on the integration of migratory and invasive signals involving cytoskeleton and extracellular matrix (ECM) remodeling [5].

Gelsolin is an actin-binding protein which severs and caps actin filaments [6], and regulates cytoskeletal turnover. Gelsolin appears to have complex roles in tumor biology, with evidence supporting its contradictory involvement in both tumor suppression as well as malignant progression. Gelsolin is reported to be down-regulated in tumors including breast [7] and lung [8] carcinomas, suggesting that loss of gelsolin promotes oncogenesis. Consistent with this, knockdown of gelsolin by small-interfering RNA (siRNA) in the immortalized human breast epithelial MCF10A cell line induced changes suggestive of epithelial-mesenchymal transformation [9]. However, there is also evidence that the re-emergence of gelsolin in tumors may promote aggressive behavior, as progressively malignant stages in cancer are associated with high gelsolin expression. Increased gelsolin expression was found to correlate with lymphatic invasion in small cell lung cancer [10] and higher tumor grade in renal cell carcinoma [11]. Intriguingly, in urothelial and oral carcinomas, gelsolin exhibited a biphasic expression profile, being down-regulated in premalignant lesions but increased in higher grade lesions [12], [13]. Furthermore, the co-expression of gelsolin with erb-B2 and epidermal growth factor receptor (EGFR) is a predictor of poor prognosis in breast cancer [14]. It is likely that the role of gelsolin differs during the course of tumor progression, and in more advanced disease gelsolin may cooperate with other oncogenic factors to accelerate progression.

Several *in vivo* and *in vitro* studies clearly indicate that gelsolin is crucial for migration and invasion in several cell types, including migration of untransformed cells such as fibroblasts and neutrophils [15], [16], as well as invasion of transformed immortalized human and canine kidney cells [17] and tumor cells including breast and prostate cancer cells across collagen and matrigel matrices [18], [19]. Gelsolin’s pro-migratory effects have been attributed to its actin-severing actions [16], [20]. However, its pro-invasive activities in epithelial cancers are unclear, and may involve a combination of mechanisms including migration and interactions with signaling proteins. Gelsolin has been shown to be a downstream effector of signaling pathways mediating invasion, including Ras and Rac GTPases, as well as phosphotidylinositol 3-kinase (PI3K) [17], [20]. Gelsolin also facilitates osteoclast podosome formation [21], and associates with the oncogenic tyrosine kinase Src in these structures [22]. Podosomes are rich in actin and mediate dynamic cell-matrix adhesion and ECM remodeling [23], [24]. Although there is now a pool of convincing evidence linking gelsolin to invasion [17], [18], [19], there is little insight (beyond gelsolin’s role in actin dynamics) on the mechanisms downstream of gelsolin leading to invasion. Previous studies have correlated the expression of actin-associated proteins such as cortactin and Lim Kinase-1 (LIMK1) with protease secretion [25], [26], and it is unknown whether gelsolin also modulates the proteolytic machinery to induce invasion. This study aims to address the gap in knowledge between gelsolin and the matrix degradation process during cancer cell invasion.

We investigated the influence of gelsolin on colorectal tumor cell dissemination and the mechanisms underlying its pro-invasive activity. Immunohistochemical (IHC) analysis showed prominent gelsolin expression along the tumor borders of both primary human colon tumors and liver metastases. The effects of gelsolin in human colorectal tumor cells were examined by inducing gelsolin overexpression as well as silencing with siRNA. Microarray analysis and quantitative PCR in these models indicated that gelsolin modulates the expression of several invasion-related genes in the urokinase-type plasminogen activator (uPA) cascade, resulting in activation of plasmin, a potent matrix degradation protease [27]. uPA and its receptor uPAR were further determined to be crucial for gelsolin-dependent invasion in colorectal tumor cells. Our work thus elucidates a novel role for gelsolin in colorectal tumor dissemination, by modulation of the uPA cascade which is crucial for invasion.

## Results

### Gelsolin Expression is Prominent at the Invasive Front of Colorectal Tumors

We analyzed the expression of gelsolin by IHC in 24 primary colorectal tumors and 26 colorectal liver metastases as well as 15 normal tissues from the surgical margins of clearance. Gelsolin expression in tumor tissues as well as the adjacent normal tissues was scored for intensity of staining (scale 0–3) and proportion of tumor positivity (scale 0–3). Primary antibody exclusion as well as mouse IgG were included as negative controls (Figure S1). In the adjacent normal colonic mucosa, the expression was prominent at the surface epithelium which comprises absorptive cells but weakly expressed in goblet cells (Figure S2). In positively-stained mucosal cells, gelsolin was present in the cytoplasm and nuclear staining was generally observed in a small proportion of cells. Gelsolin was highly expressed in myocytes of the muscularis propria and in vessel walls, consistent with previous findings [28], [29], as well as lymphoid cells.

**Figure 1:**
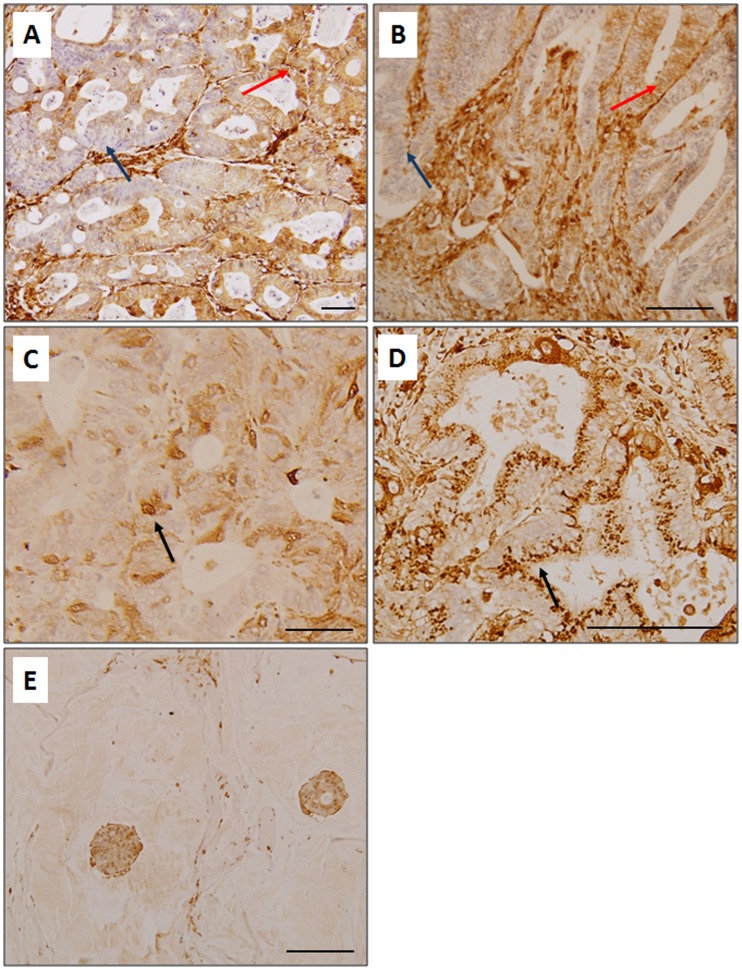
Gelsolin immunohistochemistry in human colorectal carcinoma tissues. Gelsolin is heterogeneously expressed in a number of primary tumors (A) and liver metastases (B), with islands of low (*arrowed in blue*) and high (*arrowed in red*) expression observed within a tumor. Gelsolin expression is mainly cytoplasmic but occasionally, nuclear (C) and perinuclear (D) staining are detected (*arrowed*). Gelsolin is strongly expressed in a mucinous adenocarcinoma (E) and in stroma (A, B).

**Figure 2:**
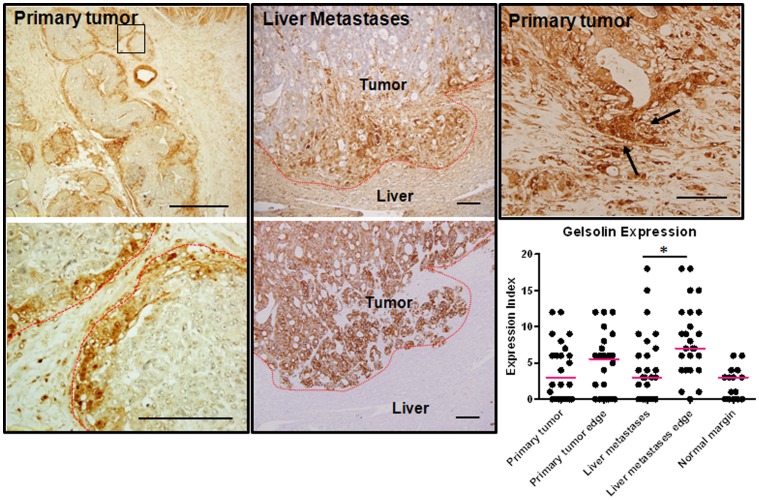
Gelsolin expression is prominent at the invasive front of human colorectal cancer tumors. Gelsolin expression is high along the tumor periphery in primary tumors and liver metastases. The tumor periphery is outlined as red dotted line. A magnified view of a region of primary tumor edge (boxed) is shown on the bottom panel. The invading liver metastases shown are confirmed by cytokeratin stain, AE1/3, on an adjacent slide (bottom middle panel). Increased gelsolin expression was also detected in less-differentiated tumor cells which appeared to be breaking away from well-formed glandular structures (arrowed, top right panel). Gelsolin expression were scored and represented in the scatter dot plot (bottom). The median scores are represented by the red horizontal bars. Mann-whitney test was to compare the gelsolin expression score between the main tumor bulk and their periphery. Bar: 50 µm.

We found gelsolin expression to be heterogeneously expressed in the matched primary tumors and liver metastases, with regions of low and high expression seen within a tumor. Gelsolin was detectable in the cytoplasm as well as the nuclei of tumor cells (Figure 1). Emerging evidence supports the importance of identifying changes within specific tumor populations, such as those at the infiltrating borders which are involved in tumor invasion and metastasis [30]. We therefore analyzed the pattern of gelsolin expression at the tumor borders compared to the tumor bulk, as these populations are potentially disseminative. In order to define the infiltrative tumor borders, adjacent sections of liver metastases were also stained with the pan-cytokeratin stain, AE1/3, which identifies tumor cells of epithelial origins. Gelsolin expression was pronounced along the tumor borders compared to tumor bulk in both primary tumors and liver metastases (Figure 2). In liver metastases, gelsolin expression was significantly higher in the tumor borders compared to the main tumor bulk (p = 0.0075, Mann-Whitney test). Interestingly we also observed high gelsolin expression in infiltrating clusters of less-differentiated cells, some of which appeared to be breaking away from well-formed glandular structures, supporting the hypothesis that gelsolin is involved in colorectal tumor cell invasion and dissemination. Our data suggests that gelsolin is further upregulated along the metastatic tumor borders and may promote secondary spread of colorectal cancer cells within the primary and secondary host tissues. Gelsolin expression was also determined in a panel of commercially-available colorectal tumor cell lines derived from primary tumors (HCT116, HT29, WiDr, RCM-1, RKO, Caco-2, SW837, SW480, SW403, DLD-1, LS513) as well as those obtained from metastatic lymph node (SW620) and ascites (COLO201 and COLO205). Among the commercially-available cell lines, the ascites-derived COLO201 and COLO205 and the primary tumor cell lines DLD-1 and LS513 expressed the highest levels of gelsolin (Figure S3).

### Gelsolin Promotes Migration and Invasion of Colorectal Tumor Cells

**Figure 3:**
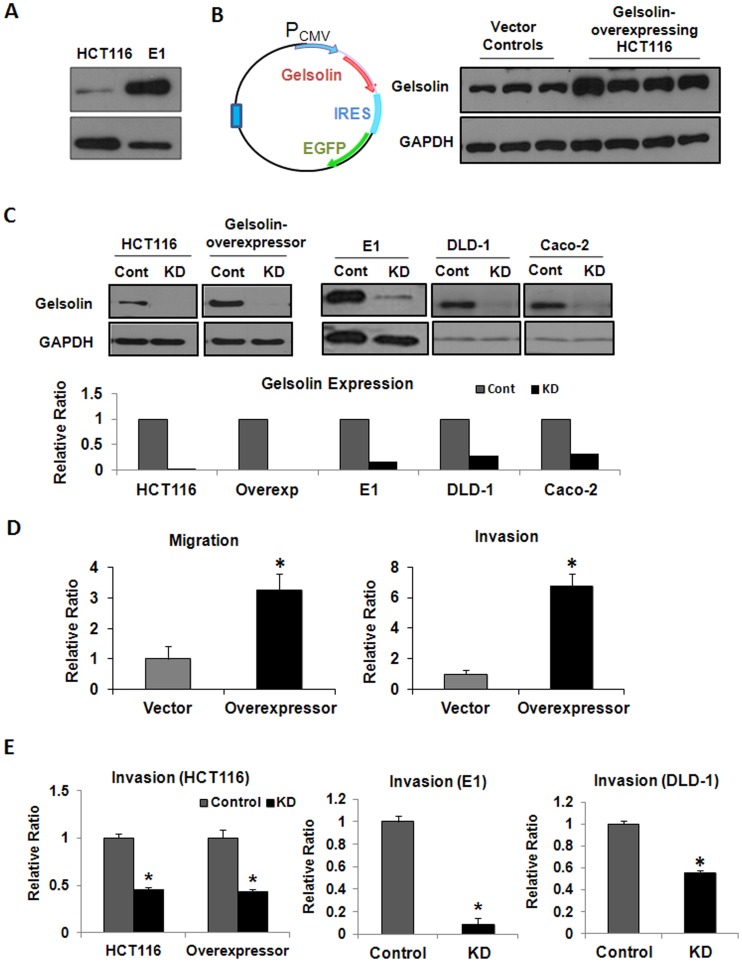
Gelsolin promotes migration and invasion of colorectal cancer cells. (**A**) Endogenous gelsolin level was increased in an *in vivo*-derived metastatic variant of HCT116, E1, which was described in [31]. (**B**) Gelsolin overexpression plasmid was constructed by cloning human cytoplasmic gelsolin cDNA into pIRES2-EGFP vector (*left*). HCT116 cells were either transfected with gelsolin-pIRES2-EGFP plasmid or empty pIRES2-EGFP plasmid to establish stable gelsolin-overexpressing cell lines and vector control cell lines respectively. Western blot analysis confirmed increased gelsolin expression in HCT116 cell lines stably-transfected with the gelsolin-overexpression plasmid compared to those transfected with control plasmid. (**C**) Western blot showing transient gelsolin siRNA knockdown (KD) at 3 days in wild-type HCT116 and gelsolin-overexpressing HCT116 cells, E1, as well as other colorectal cancer cell lines DLD-1 and Caco-2 cells. Control cells (Cont) were treated with control siRNA. (**D**) Increased gelsolin in HCT116 enhanced tumor cell migration and invasion. The migration of gelsolin-overexpressing HCT116 cells through uncoated transwells and invasion through matrigel-coated transwells were ascertained over 48 hours, and was observed to be increased compared to vector control cells. (**E**) siRNA downregulation of gelsolin abrogates invasion. Gelsolin knockdown significantly reduced invasion of HCT116 and gelsolin-overexpressing HCT116 through matrigel. A requirement for gelsolin in invasion was also observed in E1 and DLD-1 cells. All data shown are the mean ± standard error of duplicate measurements and are representative of at least three independent experiments. *P<0.05.

The oncogenic potential of gelsolin in colorectal tumor cells was investigated by modulating gelsolin levels using gelsolin overexpression and siRNA knockdown. Using the technique of in-vivo passaging through athymic nude mice, we had previously established the highly metastatic colon carcinoma E1 cell line from the poorly-metastatic HCT116 cell line [31]. HCT116 was observed to express intermediate levels of gelsolin in a panel of colorectal carcinoma cell lines investigated (Figure S3). Consistent with our hypothesis that there exists a correlation between metastatic potential and gelsolin levels, E1, the metastatic variant, expressed increased gelsolin levels compared to HCT116 (Figure 3A). To further investigate this, we overexpressed gelsolin in HCT116 cells and performed functional assays. Overexpression of gelsolin in the HCT116 was induced by stable transfection with the pIRES2-EGFP plasmid encoding human cytoplasmic gelsolin cDNA (Figure 3B; refer to Figure S4 for the cloned sequence of gelsolin cDNA). Control HCT116 cells were generated by transfection with the empty pIRES2-EGFP plasmid. In addition gelsolin expression was reduced using siRNA knockdown in several colorectal tumor cell lines (HCT116 and its metastatic variant E1, gelsolin-overexpressing HCT116, DLD-1 and Caco-2), and compared to cells transfected with control siRNA. As shown in Figure 3C, gelsolin was greatly reduced after gelsolin siRNA treatment in these cell lines. The effects of gelsolin overexpression and knockdown in colorectal tumor cells were determined as described in the sections below.

Gelsolin overexpression in HCT116 augmented migration through uncoated transwells and invasion through matrigel-coated transwells (Figure 3D). The invasiveness of the stably-transfected gelsolin-overexpressing HCT116 cells as well as wild-type HCT116 cells was significantly decreased by siRNA knockdown of gelsolin expression, indicating a reliance on gelsolin for invasion (Figure 3E). The pro-invasive role of gelsolin was consistently demonstrated in E1 and DLD-1 cells, where reduction of gelsolin by siRNA significantly attenuated invasion. Our observations indicate that gelsolin confers invasive capacity in colorectal cancer cells, which are consistent with the reported effects of gelsolin in other types of cells [17], [32].

### Gelsolin Modulates the Expression of Genes Important for Tumor Dissemination

Although gelsolin appears to be necessary for invasive behavior, little is known about the mechanisms by which it enhances invasion. This may partly be attributable to its ability to enhance cellular motility through its influence on cytoskeletal dynamics such as when lamellipodia are formed [33]. However, invasion is a complex process dependent on multiple contributory factors besides motility [5]. To further elucidate the downstream mechanisms by which gelsolin induces invasion in colorectal tumor cells, microarray analysis was performed to screen for potential genes that are differentially expressed when gelsolin levels are increased. The gene expression profiles of four stable gelsolin-overexpressing HCT116 clones were compared with two vector control HCT116 clones. The gelsolin-overexpressing clones expressed a consistent pattern of differential gene expression, suggesting that gelsolin induced specific changes in gene expression of HCT116 cells (Figure S5A). In total, we identified 469 genes with an average alteration in expression level of at least two-fold. Using the Gene Ontology functional annotation under DAVID Bioinformation Resources, genes were classified into their respective biological processes, including cell differentiation, cell motility and regulation of cell adhesion (Figure S5B). The representative table of genes modulated by gelsolin is supplied in Table S1.

### Gelsolin Modulates the Expression of Invasion-associated Urokinase-type Plasminogen Activator (uPA)

**Figure 4:**
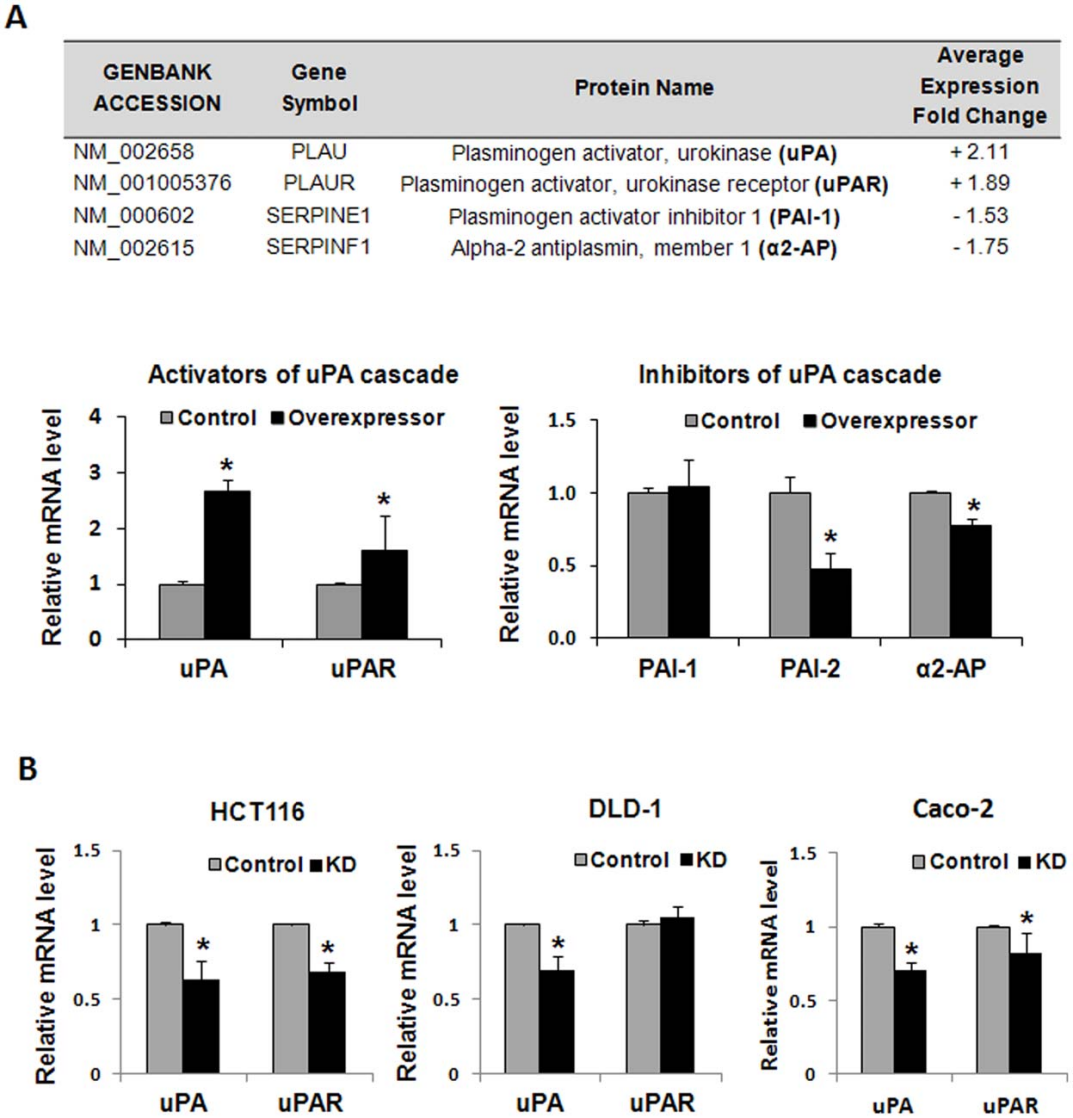
Gelsolin modulates the expression of invasion-associated urokinase-type plasminogen activator (uPA) cascade in colorectal cancer cells. (**A**) uPA was detected by microarray analysis to be differentially regulated by gelsolin overexpression. The average gene expression fold changes in the gelsolin-overexpressing clones compared to vector control clones are shown. The gene expression was verified by real-time PCR, and indicated that increased gelsolin upregulated uPA and uPAR mRNA, and decreased mRNA expression of endogenous inhibitors of uPA and plasmin, PAI-2 and α2-AP, respectively. The relative mRNA levels were normalized to vector control cells. (**B**) siRNA knockdown of gelsolin reduced uPA levels in colorectal cancer cells. Real-time PCR showing the relative mRNA expression of uPA and uPAR in HCT116, DLD-1 and Caco-2 cells after treatment with gelsolin siRNA as compared to control siRNA. All data shown are the mean ± standard error of triplicate measurements and are representative of at least three independent experiments. *P<0.05.

Among the invasion-associated genes identified to be induced by more than 2-fold in gelsolin-overexpressing cells is the serine protease, urokinase-type plasminogen activator (uPA), which is involved in the plasminogen activation cascade that results in the activation of a broad-spectrum protease plasmin. Although microarray analysis detected less than 2-fold changes in the levels of other uPA cascade genes, we included these in real-time PCR analysis as these comprise important components for regulating uPA-dependent matrix degradation. Besides uPA, the following genes were screened for mRNA expression: the uPA receptor uPAR, and known inhibitors of the uPA cascade including plasminogen activator inhibitor-1 (PAI-1), plasminogen activator inhibitor-2 (PAI-2) and α2-antiplasmin (α2-AP) [34]. Real-time PCR showed that increased levels of gelsolin in HCT116 cells upregulated the expression of uPA and uPAR, and reduced the levels of the inhibitors PAI-2 and α2-AP (Figure 4A). Consistent with observations in the overexpression studies, mRNA levels of uPA were also reduced by siRNA knockdown of gelsolin in wild-type HCT116, DLD-1 and Caco-2 cells (Figure 4B). The mRNA levels of uPAR were reduced by gelsolin siRNA-treated HCT116 and Caco-2 cells but not in DLD-1 cells. Our data indicates that uPA levels are altered by changes in gelsolin expression, and the uPA cascade is a possible mechanism through which gelsolin mediates invasion.

### Gelsolin Increases uPA Secretion by Colorectal Tumor Cells and Promotes Invasion through the uPA Cascade

**Figure 5:**
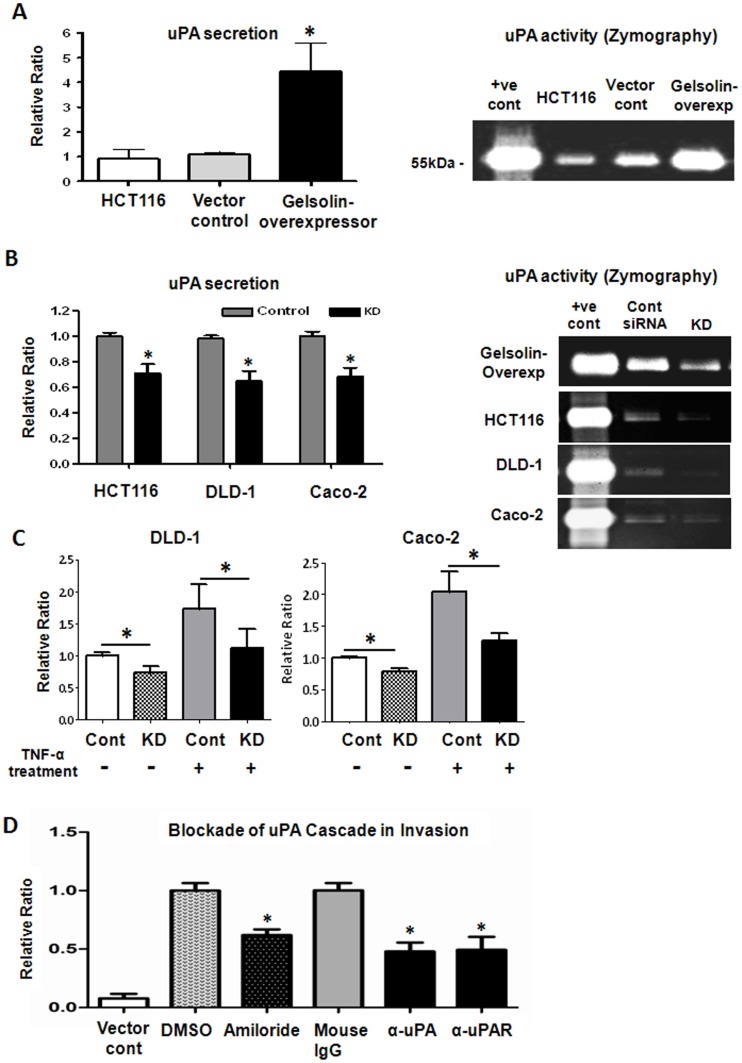
Gelsolin increases uPA secretion of colorectal tumor cells and enhances invasion via the urokinase-type plasminogen (uPA) cascade. (**A**) Increased gelsolin in HCT116 cells augmented the secretion and activity of uPA. The levels of secreted uPA by cells cultured for 48 hours in serum-free conditions were detected in the supernatant using ELISA. Gelsolin-overexpressing HCT116 cells secreted significantly higher uPA levels compared to control cells (*left*), which correlated with higher uPA enzymatic activity, as evident from uPA zymographic analysis using 16-hour serum-free conditioned media of cells (*right*). (**B**) Knockdown of gelsolin (KD) reduced the secretion of uPA. Gelsolin-overexpressing HCT116, wildtype HCT116, DLD-1 and Caco-2 cells were treated with gelsolin siRNA or control siRNA for 24 hours prior to culture under serum-free conditions for a further 48 hours. The levels of secreted uPA were detected using ELISA (*left*). Zymographic analysis indicated that uPA activity in the 3-hour conditioned media of the colorectal tumor cells was reduced 48 hours after gelsolin siRNA knockdown treatment (*right*). (**C**) Gelsolin expression affects TNF-α-stimulated uPA secretion in colorectal cancer cells. DLD-1 and Caco-2 cells were treated with gelsolin siRNA (KD) or control siRNA (Cont) for at least 24 hours and serum-starved overnight prior to incubation with 5 ng/mL (DLD-1) and 10 ng/mL (Caco-2) TNF-α for 24 hours. The levels of secreted uPA in the supernatant of cultured cells were detected using ELISA. uPA secretion was effectively stimulated by TNF-α in DLD-1 and Caco-2. However, siRNA knockdown of gelsolin significantly attentuated TNFα-stimulated uPA levels in the colorectal cancer cell lines. (**D**) Inhibition of uPA cascade attenuates the invasion of gelsolin-overexpressing HCT116 cells through matrigel. Gelsolin-overexpressing cells were treated with either 50 µM amiloride, 200 µg/mL of function-blocking anti-uPA or 80 µg/mL of anti-uPAR antibodies and examined for changes in invasive potential through matrigel. DMSO treatment and mouse IgG antibody at 200 µg/mL were used as controls to amiloride and the function-blocking antibodies and respectively. Untreated vector control HCT116 was included and normalized to DMSO vehicle control. The anti-uPA and anti-uPAR as well as amiloride treatments significantly attenuated invasion of gelsolin-overexpressing HCT116 cells, indicating that the enhanced invasiveness induced by gelsolin is mediated through the uPA cascade. All data shown are the mean ± standard error of triplicate (ELISA) and duplicate (invasion assay) measurements and are representative of at least two independent experiments. P<0.05 (student’s t test).

In the plasminogen activator cascade, the secretion of uPA is required to activate the proenzyme plasminogen to active plasmin in the extracellular environment. We investigated the levels of secreted uPA protein in the conditioned media of gelsolin-overexpressing colorectal cancer cells obtained after 48 hours of serum-deprivation, using enzyme-linked immunosorbent assays (ELISA). Consistent with the increase in uPA gene expression, we also detected significantly increased uPA secretion in gelsolin-overexpressing HCT116 compared to control cells (Figure 5A). We examined the effects of gelsolin-induced uPA expression on matrix degradation using zymographic analysis. Conditioned media of the cells were loaded and electrophoresed in zymographic gels containing plasminogen and fibrinogen for analysis of uPA activity. Gelsolin-overexpressing HCT116 cells displayed greater uPA activity in comparison to vector control and wildtype HCT116, as evident from the enhanced lysis zones (Figure 5A). The increased proteolytic ability of gelsolin-overexpressing cells, as ascertained by our zymographic analysis, corroborates with earlier observations from the transwell invasion assay which showed that gelsolin conferred invasiveness in colorectal cancer cells (Figure 3D–E). Conversely, siRNA knockdown of gelsolin in the colorectal tumor cell lines HCT116, DLD-1 and Caco-2 significantly reduced the level of secreted uPA and its proteolytic activity (Figure 5B). Our data suggests that gelsolin modulates uPA activity on the extracellular matrix by influencing uPA expression and its secretion.

The cytokine, tumor necrosis factor alpha (TNF-α), is produced by several types of tumors and has been reported to stimulate uPA production [35] and enhance invasion [36]. As shown in Figure 5C, treatment with TNF-α for 24 hours enhanced uPA secretion in DLD-1 and Caco-2 cells. However when gelsolin expression was reduced by siRNA knockdown, the increases in uPA secretion induced by TNF-α were significantly attenuated to levels similar to that in control cells. Our data shows that TNF-α induced uPA secretion is attenuated in the absence of gelsolin. However, it is currently unclear how gelsolin modulates uPA secretion in response to TNF-α stimulation.

The involvement of the uPA cascade in the increased invasiveness of gelsolin-overexpressors was further confirmed by inhibiting uPA function and examining the effects on invasion through matrigel. The highly invasive gelsolin-overexpressing HCT116 cells were treated with function-blocking antibodies against either uPA or uPAR, or by treatment with the uPA inhibitor amiloride [37]. Control experiments were conducted using isotype-control mouse IgG and vehicle control dimethyl sulfoxide (DMSO), respectively. Blockade of the uPA cascade using either anti-uPA or anti-uPAR antibody, or amiloride significantly attenuated the invasiveness of gelsolin-overexpressing HCT116 cells across matrigel, by 40% to 55% (Figure 5D). Our data indicated that gelsolin enhances invasion through the uPA activator cascade, by promoting uPA secretion which can enhance matrix degradation.

Apart from members of the uPA cascade, the genes of various other proteases or related proteins such as metalloproteinase-7 (MMP7) and TIMP2 were also modulated by gelsolin overexpression in HCT116 (Table S1). Since tumor cells may secrete different proteases during the course of dissemination, we investigated whether the gelsolin-mediated invasion may involve, besides uPA, members of the MMP family. Gelsolin-overexpressing cells were subjected to similar transwell invasion assays following treatment with either the synthetic pan-MMP inhibitor GM6001 (which blocks MMP-1, -2, -3, -8 and -9 activities) or other specific inhibitors to MMP-2, MMP-2/−9, MMP-3, MMP-7 and MMP-8. Pan-MMP inhibition using GM6001, as well as MMP-2 inhibition reduced the invasiveness of cells by about 20%, although the results were not statistically significant (Figure S6). The simultaneous inhibition of MMP-2/MMP-9 showed similar effect to inhibition of MMP-2 alone, suggesting that MMP-9 played little or no role in the invasion of gelsolin-overexpressing cells. There was however no significant increase in the MMP-2 activity in gelsolin-overexpressing cells compared to vector control and HCT116 based on gelatin zymography (Data not shown). Inhibition of MMP-3, MMP-7 and MMP-8 had no effect on invasion (Figure S6). Taken together our data indicated that in addition to its pro-migratory role, gelsolin promotes invasion in the colorectal carcinoma cells predominantly via increased expression and secretion of uPA.

## Discussion

In this study, we show that invading populations of tumor cells enriched in gelsolin are found in both primary as well as metastatic human colorectal cancers. This is consistent with earlier observations of prominent gelsolin expression along the invasive front of liver metastases, in contrast to its low expression in primary colorectal adenocarcinomas [28]. The significance of “leading cells” at the invasive front of tumors in promoting tumor spread has been highlighted in a number of studies. Cells at the invasive edge acquire molecular changes such as increased expression of matrix-digesting proteases and integrins for matrix-remodelling [38], [39], which may be accompanied by a switch towards a mesenchymal-like, dedifferentiated phenotype. These changes in leading cells at the invasive front drive proteases- and force-mediated matrix remodeling which pave the way for collective cell invasion by non-invasive “follower cells” [39]. Notably, the increased tumor aggressiveness has been associated with increased expression or altered localization of other actin-binding proteins such as β-catenin [30], actinin-4 [40], cortactin [41] and fascin [42] at the invasive region of tumors. We have demonstrated here that gelsolin is crucial for the invasive behavior of colorectal tumor cells. Gelsolin has previously been shown to be important for migration of fibroblasts and invasion of other tumor cells, attributable partly to its actin-depolymerizing effects. However, invasion involves the coordination of multiple mechanisms, which include migration and ECM degradation. It is conceivable that the effect of gelsolin siRNA knockdown on invasion through matrigel may be partially attributed to a reduced migratory capacity of cells. However, the pro-invasive role of gelsolin via matrix degradation was reaffirmed by zymographic analyses, which enabled the detection of proteolytic activity of secreted proteases. Our work shows that, in addition to previous work highlighting gelsolin’s roles in driving lamellipodia protrusion and turnover in migration [43], gelsolin also confers invasive properties via its ability to modulate the expression of well-known mediators of tumor invasion - genes involved in the uPA cascade which degrade the ECM.

The uPA cascade is initiated when secreted uPA from cancer cells [44] or stromal components [45] binds to its receptor uPAR and converts the inactive plasminogen to active plasmin by proteolytic cleavage. Plasmin promotes remodeling of ECM by direct proteolysis, or indirectly through activation of MMPs [46], [47]. The uPA system is implicated as a major factor leading to aggressive tumor behaviour, as it promotes invasion and metastasis in several tumor types including colorectal cancer [48], [49]. uPA and uPAR activities contribute to proteolysis of ECM at the invasive front of tumors [50], and elevated levels in colorectal tumors correlate with tumor progression and poor survival [51], [52].

Our findings revealed the ability of gelsolin to modulate uPA gene expression and secretion. The roles of cytoskeletal proteins in regulation of gene expression is an emerging field of interest, as it is now clear that the nuclear cytoskeleton participates in several transcriptional regulatory processes [53]. Indeed, actin-binding proteins, including gelsolin and its family members have prominent roles in mediating nuclear receptor-directed transcription [54]. Gelsolin was previously reported to physically interact with androgen receptor (AR) and enhance AR transcriptional activity, [55]. More recently, gelsolin was found to be a key determinant for the assembly and/or stability of estrogen receptor nucleus complexes [56]. Interestingly, the nuclear import of CapG, another gelsolin family member, is suggested to interfere with chromosome condensation [53] and promote invasion into collagen [57]. It is unknown if gelsolin also regulates non-nuclear receptor-dependent transcription, though its presence in the nuclei of colorectal tumor tissues suggests wider roles for nuclear-associated gelsolin. As yet, it is unclear how gelsolin regulates uPA gene expression - this would be an interesting avenue for further exploration into gelsolin’s roles in transcriptional regulation of invasion genes.

Besides modulating the mRNA levels of uPA, we also found that gelsolin regulates uPA secretion by colorectal cancer cells. Increased circulating levels of plasma uPA have previously been associated with advanced cancers including colorectal [58] and prostate cancers [59]. Very recently gelsolin was reported to regulate insulin exocytosis through its direct interaction with syntaxin 4, a plasma membrane protein which mediates docking of transport vesicles [60]. The expression of other actin-associated proteins, such as cortactin and LIMK1, has also been reported to correlate with the secretion of matrix proteases including MMPs [25], [26], [61]. LIMK1 in particular, which regulates the activities of actin-depolymerizing factor (ADF/cofilin), has also been shown to interact with membrane type 1 (MT1)-MMP and regulate its vesicular trafficking [25]. It is possible that gelsolin may also participate in the transport of vesicles containing uPA or other proteases through its influence on F-actin dynamics which is essential for vesicular trafficking. Future studies on the role of gelsolin in the secretory pathway of proteases such as uPA would yield a clearer picture of the integration of the distinct but synergistic cellular processes mediated by gelsolin to promote invasion. Nevertheless, the revelation of gelsolin’s function in regulating uPA expression and secretion in colorectal cancer invasion provides further insight into the mechanisms behind gelsolin’s oncogenic role, and lends support to the multiple roles of actin cytoskeletal proteins in promoting cancer cell dissemination.

In the current study we also investigated the contribution of a number of MMPs in the gelsolin-mediated invasion in colorectal cancer cells. Inhibition of the gelatinase MMP-2 alone led to a slight decrease in invasion but not the inhibition of MMP-3, -7, -8 and -9 in gelsolin-overexpressing cells. Treatment with GM6001 which inhibits MMP-1 in addition to MMP-2, MMP-3, MMP-8 and MMP-9 reduced invasion to a small extent but the results were not statistically significant. Interestingly proMMP-2 was reported to be activated by plasmin [47], [62], which may increase the pool of active MMP-2. Although plasmin can also activate the other gelatinase proMMP-9, it is pertinent to add that plasmin is not an efficient activator of proMMP-9 [46]. Nevertheless, the balance between MMPs and their endogenous inhibitors, tissue inhibitor of metalloproteinases (TIMPs), is critical in determining the net effect on matrix degradation. Our unpublished data indicated that TIMP-2 was increased in gelsolin-overexpressing HCT116 cells, with the direct implication being that the matrix-degrading effects of MMPs can be counter-balanced. Of note, literature also reveals paradoxical, pro-tumor roles of TIMPs in cancer biology [63]. Due to the complexity of the biological activities of MMPs and TIMPs, it is not within the scope of this study to look into the interactions between MMPs and TIMPs in detail. Moreover the evidence we presented in this manuscript suggests that the uPA cascade is a major downstream pathway by which gelsolin induce matrix degradation.

Together with the well-established functions of gelsolin in cytoskeletal dynamics, our findings implicate gelsolin as a regulatory determinant of the uPA cascade, with significant impact on colorectal cancer invasion. The potential contributions of gelsolin towards the further spread of tumor cells from liver metastases warrant further investigations into the roles of cytoskeletal proteins in metastatic disease. Further dissection of the mechanisms by which gelsolin and other cytoskeletal proteins regulate invasive pathways could contribute towards the understanding of how cancer progresses, and the development of effective strategies which counteract its spread.

## Materials and Methods

### Cell Lines and Reagents

HCT116, HT29, WiDr, RCM-1, RKO, Caco-2, SW837, SW480, SW403, SW620, DLD-1, LS513, COLO201 and COLO205 are human colon cancer cell lines obtained from ATCC. The *in vivo*-derived metastatic E1 cell line was developed from HCT116, as previously described [31]. HCT116, E1, HT29 and WiDr were cultured in McCoy’s 5A modified medium; Caco-2, SW480, SW837, SW403 in Dulbecco’s Modified Eagle’s Medium and DLD-1, RKO, LS513, RCM-1, COLO201, COLO205 in RPMI 1640 (All media from Sigma-Aldrich). All media were supplemented with 10% fetal bovine serum (FBS) (Hyclone). Stable HCT116 cell lines overexpressing gelsolin and empty vector control cell lines were grown using the McCoy’s 5A medium with addition of 500 µg/ml Geneticin (Gibco). Cells were maintained at 37°C in a humidified incubated with 5% CO2. Antibodies used include mouse antibodies against human gelsolin (Abcam), β-actin (Sigma-Aldrich), GAPDH (Santa Cruz Biotechnology), pan-cytokeratin (AE1/3) (Dako), uPA (America Diagnostica), uPAR, mouse IgG1 and goat IgG (R&D Systems). Secondary antibody goat anti-mouse IgG conjugated with HRP (Santa Cruz Biotechnology) was used. Metalloproteinase (MMP) inhibitors include GM6001, MMP-2 Inhibitor I, MMP-2/MMP-9 Inhibitor IV, MMP-3 Inhibitor II, MMP-8 Inhibitor I (Calbiochem) as well as anti-MMP7 antibody (R&D Systems).

### Construction and Transfection of Gelsolin-overexpression Plasmids

A coding sequence of human gelsolin was amplified from a PMW172 expression plasmid containing human cytoplasmic gelsolin cDNA, by polymerase chain reaction (PCR) using the forward primer 5′CG GAA TTC ATG GTG GTG GAA CAC CCC GAG TTC 3′ and reverse primer 5′ CG CCG CGG TCA GGC AGC CAG CTC AGC CAT GGC 3′, followed by cloning of gelsolin insert into pIRES2-EGFP vector (Becton Dickinson) at Eco RI and Sac II enzyme restriction sites. The resulting vectors were transformed into competent *Escherichia* coli XL1B MRF’ cells (Stratagene) and clones were selected based on kanamycin resistance. Sequencing of the cloned gelsolin cDNA verified that the cDNA sequence encodes the human cytoplasmic gelsolin protein (Swiss-Prot accession P06396). To generate stable cell lines, gelsolin- pIRES2-EGFP vector or empty vector were transfected into HCT116 using FuGENE 6 (Promega) and selected using 500 µg/ml G418.

### Immunohistochemistry

24 primary colorectal tumors and 26 colorectal liver metastases tissues as well as 15 adjacent normal colonic tissues were obtained for use from the Department of Pathology, National University Hospital, Singapore, with approval from the National University of Singapore Institutional Review Board. Briefly, the 4 µm-thick tissue specimens were deparaffinized, boiled in citric acid, and treated with hydrogen peroxide, before incubation with anti-gelsolin primary antibody overnight at 4°C, followed by incubation with polymer-horse radish peroxidase-conjugated secondary antibody at room temperature. Sections were developed with diaminobenzidine (DAB) and counterstained with hematoxylin. Images were acquired using Olympus BX43F microscope equipped with a DP70 camera. Gelsolin staining in the muscularis propria and stroma [28], [29] were used as internal positive controls. The intensity of staining was graded from 0 (undetectable) to 3 (intense staining), whilst the proportion of positive staining tumor cells within a tissue was scored from 0 to 3 where: 1 = <30%, 2 = 30–60%, 3 = >60% of tumor cells identified. The staining score was expressed as the product of intensity of staining and proportion of tumor positivity. Both cytoplasmic and nuclear staining were scored and summed. The maximum possible score for a sample is therefore 18, the sum of the maximum cytoplasmic (9) and nuclear scores (9).

### siRNA Transfection

10 nM of the Stealth siRNA of the sequence 5′ AAA CGU CCA AUC UUG UUG GAG CAG G 3′ (Invitrogen), complexed with lipofectamine, was used to silence gelsolin expression in cells. Medium GC control siRNA which matched the GC content of the gelsolin siRNA used and no-siRNA treatment were included as controls. Cells were harvested at 48 or 72 hours, and treated as used in other assays as described.

### Matrigel Invasion Assay

The transwell assay was carried out as previously described [31]. 5×10^4^ cells were seeded for HCT116 and E1 cell lines and 3×10^4^ cells for DLD-1, for 48 hours. 10% FBS was used as chemoattractant in the bottom transwell chamber. For function-blocking experiments, cells were seeded with 200 µg/mL anti-uPA or 80 µg/mL anti-uPAR (American Diagnostica). For MMP inhibition treatments, 0.15×10^6^ gelsolin-overexpressing HCT116 cells were seeded for 24 hours with 40 µM of MMP inhibitor (either GM6001 or MMP-2, MMP-2/-9, MMP-3 inhibitor), except for MMP-8 inhibitor which was used at 1 µM. Mouse IgG1and goat IgG were included as controls for anti-uPA/anti-uPAR and anti-MMP7 respectively whereas the vehicle control dimethyl sulfoxide (DMSO) was used alongside with the remaining MMP inhibitor treatments. The same protocol was used for migration studies but without the addition of matrigel coating on the transwell membrane.

### ELISA

For measurement of secreted uPA under standard culture condition, cells were incubated in serum-free medium for 48 hours before harvest of culture supernatant for assay. For stimulated conditions, cells were serum-starved overnight prior to incubation with either 5 or 10 ng/mL of human recombinant TNF-α (PeproTech) in fresh serum-free media for 24 hours. As a control for TNF-α stimulation, cells were incubated with serum-free media in parallel. All harvested cell culture supernatants were kept at −20°C until analysis by ELISA. For measurement of secreted uPA under gelsolin-knockdown conditions, cells were treated with siRNA before the serum-starvation step on the following day. Levels of uPA present in the neat supernatants were determined by the human Duoset ELISA Development Kit (R&D Systems), according to the manufacturer’s protocol.

### Microarray Analysis

Microarray analysis was performed on the wild-type HCT116 cell line as well as stably-transfected HCT116 cells including four gelsolin-overexpressing cell lines and two empty vector-transfected control cell lines using Sentrix HumanRef-8 Beadchips (Illumina), according to the protocol outlined in the Illumina technical manual. The arrays were scanned using Ilumina BeadArray Reader and analyzed using BeadStudio version 3.1.3.0. For each cell line, duplicates from two independent harvests were profiled. The absolute data was exported into GeneSpring GX v7.3 software (Silicon genetics) for further analysis. The measurements on each chip were first divided by the 50^th^ percentile value, after which the value obtained for each gene was normalized to the average baseline median value of the control samples. One-way ANOVA approach was used to identify differentially-expressed genes and genes showing an average of at least a two-fold change in expression level in the four gelsolin-overexpressing samples. The output was then functionally annotated via Gene Ontology functional annotation under DAVID Bioinformation Resources (http://david.abcc.ncifcrf.gov/). The microarray data reported here are described in accordance with MIAME guidelines, and is accessible at Gene Expression Omnibus (GEO) database [64] through GEO Series accession number GSE36588 (http://www.ncbi.nlm.nih.gov/geo/query/acc.cgi?acc=GSE36588).

### Real Time Quantitative RT-PCR

Real Time PCR was used to validate gene expression change from microarray data. 0.5–1.4 mg of cDNA was used for reverse-transcription, carried out on the ABI 7300 Real time PCR system. The thermal cycling conditions were as follows: one cycle at 95°C for 10 minutes, followed by 40 cycles of denaturation at 95°C for 15 seconds and annealing extension at 60°C for 1 minute. The primers used included uPA (Hs01547054_m1), uPAR (Hs00182181_m1), PAI-1 (Hs00167155_m1), PAI-2 (Hs00234032_m1) and α2-antiplasmin (Hs00171467_m1). GAPDH (Hs99999905_m1) was included as the internal control. All reagents are from Applied Biosystems.

### Western Blotting

Total cell lysates were extracted using lysis buffer (6 M urea, 1% SDS, 2 M β-mercaptoethanol, 1 M Tris pH 7.4, PBS). Equivalent amounts of protein from each sample were separated on 10% SDS-PAGE gels and transferred to PVDF membranes, blocked and incubated with primary antibody overnight at 4°C followed by secondary antibody before chemiluminescence substrate detection.

### Zymography

Secreted uPA enzymatic activities were examined by zymography. Briefly, cells were grown in serum-free medium for 3 to 16 hours. The conditioned media were normalized using cellular lysate and then combined with non-reducing sample loading buffer and loaded into 10% SDS-PAGE gels containing 730 ug/mL of human fibrinogen and 20 ug/mL human plasminogen (Sigma-aldrich). After electrophoresis, gels were rinsed with distilled water and incubated with wash buffer (2.5% Triton X-100 and 50 mM Tris-HCl pH 8.0) for 1 hour, followed by incubation at 37°C with incubation buffer (0.1 M glycine buffer, pH 8.0) for 16 hours. The gels were then stained with Coomassie blue (0.05% Coomassie dye, 40% methanol, and 10% acetic acid) for 1 hour at room temperature, and destained with distilled water. Areas of lysis were expected to appear as zones of clearance against the Coomassie blue-stained background of undegraded substrate.

### Statistical Analysis

Statistical analysis was performed using Student’s t-test except for IHC gelsolin expression analysis in which Mann-Whitney test was used. Differences between sample means were considered statistically significant with P<0.05.

## Supporting Information

Figure S1 **Gelsolin immunohistochemistry in human colon tissues.** An example of gelsolin staining in adjacent liver metastases section is shown here. Negative controls, including primary antibody exclusion and IgG isotype control were included to confirm the specificity of the gelsolin antibody used. Gelsolin was consistently highly expressed in the stroma but stromal stainings were mainly undetectable in the negative control samples. Bar: 50 µm.
(TIF)

Figure S2 **Gelsolin immunohistochemistry in normal human colon tissues.** Gelsolin is prominent in surface epithelium cells (**A**, *arrowed*) and weakly expressed, or absent in goblet cells (**B, C**). Cytoplasmic labelling is predominant, with occasional nuclear localization (**C**, *arrowed*). Gelsolin is intensely expressed in muscularis propria (**D**), vessel walls (**E**) and lymphoid cells (**F**). Bar: 50 µm.
(TIF)

Figure S3 **Gelsolin immunohistochemistry in human colorectal carcinoma cell lines.** In a panel of colorectal cell lines, gelsolin levels are highest in COLO201 and COLO205, both of which were obtained from metastatic ascites. Gelsolin expression in the remaining primary tumor-derived cell lines (except SW620 which was derived from lymph node) were more varied. The graph displays normalized gelsolin expression to HCT116. β-actin was used as loading control.
(TIF)

Figure S4 **Cloned sequence of human cytoplasmic gelsolin cDNA.** Nucleotides 159 to 2354 was cloned. This sequence is a 100% match with Genbank accession #BC026033.1.
(TIF)

Figure S5 **Gelsolin modulates the expression of genes important for tumor dissemination.** **(A)** The global gene expression profile of four stable gelsolin-overexpressing HCT116 cell lines were compared against the pooled average of two vector control cell lines using microarray analysis. Each horizontal row in the cluster diagram represents a gene. Blue shades represent downregulation while red shades represent upregulation in gene expression relative to the vector control cells. All samples were assayed in independent duplicates. **(B)** Biological function classification of differentially-expressed genes from the microarray output, using Gene Ontology annotation from DAVID bioinformation resources. Genes showing an average of at least two-fold change in expression level in the four gelsolin-overexpressing clones are represented in the classification. Gelsolin modulates the genes involved in tumor dissemination including cell differentiation, cell motility and the regulation of cell adhesion.
(TIF)

Figure S6 **Gelsolin-overexpressing HCT116 may enhance invasion by MMP-2.** Gelsolin-overexpressing cells were treated with MMP inhibitors and examined for changes in invasive potential through matrigel. The pan-MMP inhibitor GM6001 as well as inhibitors to MMP-2, MMP-2/-9 and MMP-3 were used at 40 µM, MMP-8 inhibitor at 1 µM, and α-MMP7 antibody at 80 µg/mL. DMSO and goat IgG antibody were used as controls to MMP chemical inhibitors and α-MMP7 antibody treatments respectively. No significant reduction in invasion was observed in any of the MMP inhibitor treatments, although GM6001 and MMP-2, MMP-2/−9 inhibitors showed a slight reduction. All data shown are the mean ± standard error of at least duplicate measurements and are representative of at least two independent experiments.
(TIF)

Table S1 **Gelsolin modulates the expression of genes important for various cellular processes.** Genes from the microarray output were classified under various biological processes using Gene Ontology annotation from DAVID bioinformation resources. Average fold change in gene expression is determined from comparison between 4 gelsolin-overexpressing HCT116 clones and 2 vector-control HCT116 clones.
(PDF)
