# Supplementary figures and images for: Gelsolin Induces Colorectal Tumor Cell Invasion via Modulation of the Urokinase-Type Plasminogen Activator Cascade
Source: PLoS One. 2012 Aug 21;7(8):e43594. doi: 10.1371/journal.pone.0043594 (PMC3424201; doi:10.1371/journal.pone.0043594)

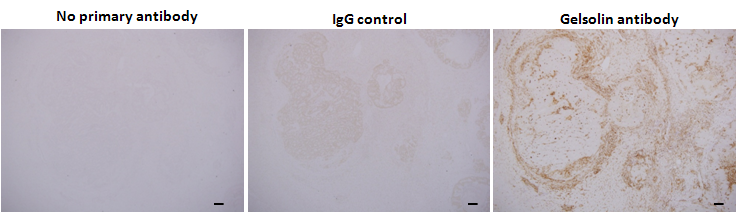

Supplement: Figure S1 — Gelsolin immunohistochemistry in human colon tissues. An example of gelsolin staining in adjacent liver metastases section is shown here. Negative controls, including primary antibody exclusion and IgG isotype control were included to confirm the specificity of the gelsolin antibody used. Gelsolin was consistently highly expressed in the stroma but stromal stainings were mainly undetectable in the negative control samples. Bar: 50 µm. (TIF) [file pone.0043594.s001.tif]

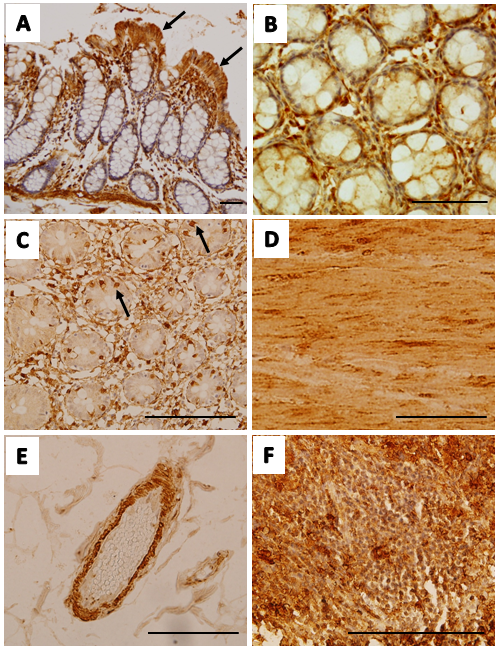

Supplement: Figure S2 — Gelsolin immunohistochemistry in normal human colon tissues. Gelsolin is prominent in surface epithelium cells (A, arrowed) and weakly expressed, or absent in goblet cells (B, C). Cytoplasmic labelling is predominant, with occasional nuclear localization (C, arrowed). Gelsolin is intensely expressed in muscularis propria (D), vessel walls (E) and lymphoid cells (F). Bar: 50 µm. (TIF) [file pone.0043594.s002.tif]

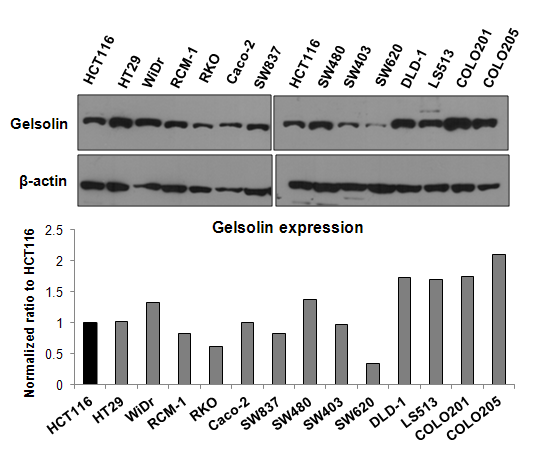

Supplement: Figure S3 — Gelsolin immunohistochemistry in human colorectal carcinoma cell lines. In a panel of colorectal cell lines, gelsolin levels are highest in COLO201 and COLO205, both of which were obtained from metastatic ascites. Gelsolin expression in the remaining primary tumor-derived cell lines (except SW620 which was derived from lymph node) were more varied. The graph displays normalized gelsolin expression to HCT116. β-actin was used as loading control. (TIF) [file pone.0043594.s003.tif]

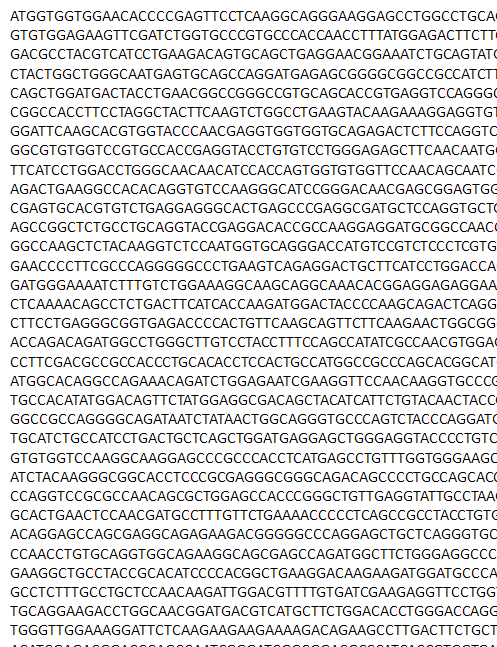

Supplement: Figure S4 — Cloned sequence of human cytoplasmic gelsolin cDNA. Nucleotides 159 to 2354 was cloned. This sequence is a 100% match with Genbank accession #BC026033.1. (TIF) [file pone.0043594.s004.tif]

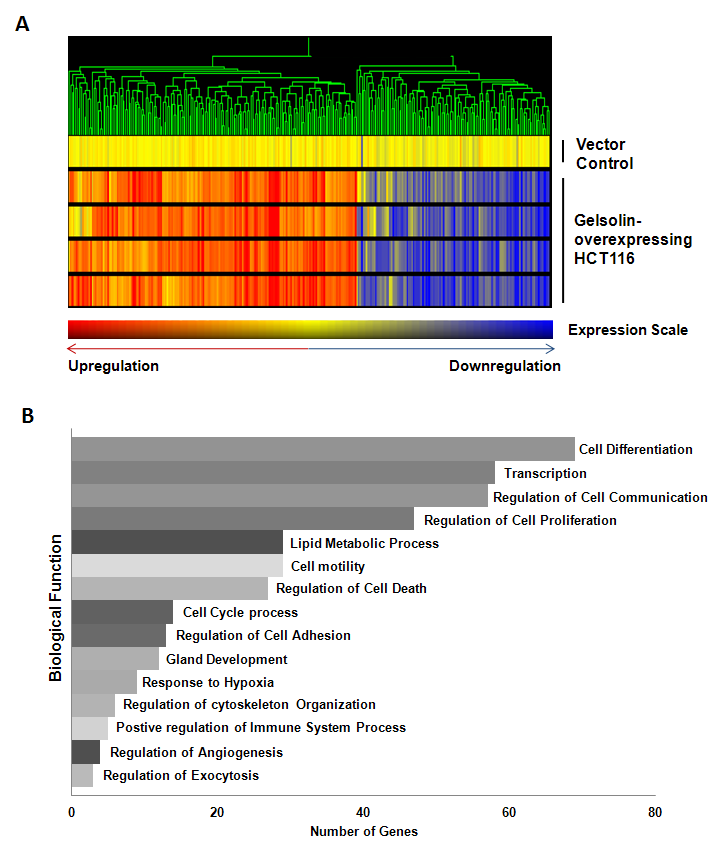

Supplement: Figure S5 — Gelsolin modulates the expression of genes important for tumor dissemination. (A) The global gene expression profile of four stable gelsolin-overexpressing HCT116 cell lines were compared against the pooled average of two vector control cell lines using microarray analysis. Each horizontal row in the cluster diagram represents a gene. Blue shades represent downregulation while red shades represent upregulation in gene expression relative to the vector control cells. All samples were assayed in independent duplicates. (B) Biological function classification of differentially-expressed genes from the microarray output, using Gene Ontology annotation from DAVID bioinformation resources. Genes showing an average of at least two-fold change in expression level in the four gelsolin-overexpressing clones are represented in the classification. Gelsolin modulates the genes involved in tumor dissemination including cell differentiation, cell motility and the regulation of cell adhesion. (TIF) [file pone.0043594.s005.tif]

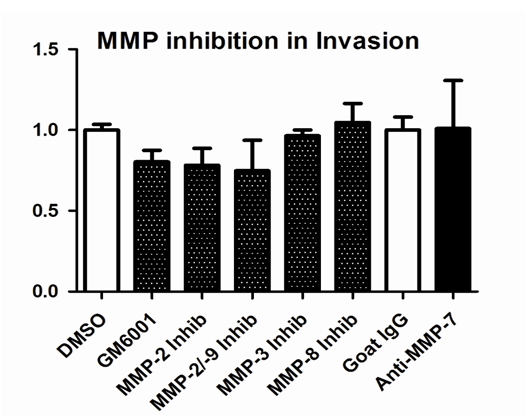

Supplement: Figure S6 — Gelsolin-overexpressing HCT116 may enhance invasion by MMP-2. Gelsolin-overexpressing cells were treated with MMP inhibitors and examined for changes in invasive potential through matrigel. The pan-MMP inhibitor GM6001 as well as inhibitors to MMP-2, MMP-2/-9 and MMP-3 were used at 40 µM, MMP-8 inhibitor at 1 µM, and α-MMP7 antibody at 80 µg/mL. DMSO and goat IgG antibody were used as controls to MMP chemical inhibitors and α-MMP7 antibody treatments respectively. No significant reduction in invasion was observed in any of the MMP inhibitor treatments, although GM6001 and MMP-2, MMP-2/−9 inhibitors showed a slight reduction. All data shown are the mean ± standard error of at least duplicate measurements and are representative of at least two independent experiments. (TIF) [file pone.0043594.s006.tif]
