# Supplementary material for: Gelsolin Induces Colorectal Tumor Cell Invasion via Modulation of the Urokinase-Type Plasminogen Activator Cascade
Source: PLoS One. 2012 Aug 21;7(8):e43594. doi: 10.1371/journal.pone.0043594 (PMC3424201; doi:10.1371/journal.pone.0043594)
Supplement: Table S1 — Gelsolin modulates the expression of genes important for various cellular processes. Genes from the microarray output were classified under various biological processes using Gene Ontology annotation from DAVID bioinformation resources. Average fold change in gene expression is determined from comparison between 4 gelsolin-overexpressing HCT116 clones and 2 vector-control HCT116 clones. (PDF) [file pone.0043594.s007.pdf]

Cell Differentiation		
Genbank Accession no.	Protein Name	Ave Fold Δ
ILMN_13615	E74-like factor 3 (ets domain transcription factor, epithelial-specific)	2.80
ILMN_21869	EPH receptor A4	2.58
ILMN_5986	G protein-coupled receptor 183	-2.15
ILMN_5586	KIT ligand	-2.57
ILMN_8384	PR domain containing 1, with ZNF domain	-2.43
ILMN_19136	RAN, member RAS oncogene family	-2.69
ILMN_15630	SATB homeobox 2	-2.25
ILMN_5130	SH2B adaptor protein 3	-4.36
ILMN_1046	TIMP metallopeptidase inhibitor 2	4.25
ILMN_28887	Zic family member 2 (odd-paired homolog, Drosophila)	-2.25
ILMN_23272	amyloid beta (A4) precursor protein	2.19
ILMN_18802	angiopoietin 2	-40.43
ILMN_20440	angiopoietin-like 4	-2.05
ILMN_27187	bone morphogenetic protein 4	-3.20
ILMN_7507, ILMN_28609	brain-derived neurotrophic factor	7.39
ILMN_1624	caveolin 1, caveolae protein, 22kDa	3.05
ILMN_5108	caveolin 2	2.86
ILMN_3374	connective tissue growth factor	3.35
ILMN_3062	cyclin-dependent kinase 6	-2.36
ILMN_10501	cystatin A (stefin A)	-7.48
ILMN_21296	cytochrome P450, family 24, subfamily A, polypeptide 1	-17.96
ILMN_27871	dedicator of cytokinesis 7	-22.54
ILMN_5440	dual specificity phosphatase 6	-5.84
ILMN_10721	early growth response 2	-2.67
ILMN_3827	ephrin-B2	-2.44
ILMN_15615	epidermal growth factor receptor (erythroblastic leukemia viral (v-erb-b) oncogene homolog, avian)	2.23
ILMN_17131	epiregulin	-2.63
ILMN_25097	epithelial membrane protein 1	3.82
ILMN_16913	ets homologous factor	5.02
ILMN_18897	fibroblast growth factor 19	-2.23
ILMN_1771	fibroblast growth factor 9 (glia-activating factor)	-3.80
ILMN_21371	filamin B, beta (actin binding protein 278)	6.82
ILMN_23624	forkhead box C1	-3.12
ILMN_22730, ILMN_11197	glutaredoxin 2	-2.11
ILMN_18641	hairy/enhancer-of-split related with YRPW motif 1	-2.36
ILMN_6829	inhibitor of DNA binding 3, dominant negative helix-loop-helix protein	-3.22
ILMN_25529	insulin-like growth factor binding protein 3	3.05

Cell Differentiation (*Continued*)		
**Genbank Accession no.**	**Protein Name**	**Ave Fold Δ**
ILMN_12662	integrin, alpha 2 (CD49B, alpha 2 subunit of VLA-2 receptor)	-4.25
ILMN_6529	keratin 19	3.35
ILMN_4021	laminin, alpha 4	-2.48
ILMN_7322	laminin, beta 1	-4.95
ILMN_3286	microtubule-actin crosslinking factor 1	-2.12
ILMN_28251, ILMN_9993	microtubule-associated protein 1B	3.00
ILMN_3041, ILMN_10370	myelin basic protein	2.50
ILMN_26696	myosin, light chain 2, regulatory, cardiac, slow	2.08
ILMN_26971	neuronal guanine nucleotide exchange factor	2.17
ILMN_17483	neuropilin 1	-2.77
ILMN_7200	nuclear receptor subfamily 2, group F, member 1	-6.66
ILMN_28405	nuclear receptor subfamily 4, group A, member 2	-2.58
ILMN_17074	ovo-like 2 (Drosophila)	-2.45
ILMN_19894	parathyroid hormone 1 receptor	2.06
ILMN_6504	periplakin	2.68
ILMN_7980	peroxisome proliferator-activated receptor gamma	-2.01
ILMN_5923	phosphoinositide-3-kinase, regulatory subunit 1 (alpha)	2.62
ILMN_27588	pre-B-cell leukemia homeobox 1	2.33
ILMN_15492	proprotein convertase subtilisin/kexin type 9	2.02
ILMN_18896	protein tyrosine phosphatase, receptor type, F	2.02
ILMN_24484	sema domain, immunoglobulin domain (Ig), short basic domain, secreted, (semaphorin) 3A	-81.44
ILMN_29382	sema domain, immunoglobulin domain (Ig), short basic domain, secreted, (semaphorin) 3C	2.03
ILMN_25026	sema domain, immunoglobulin domain (Ig), transmembrane domain (TM) and short cytoplasmic domain, (semaphorin) 4B	2.33
ILMN_11282	sema domain, transmembrane domain (TM), and cytoplasmic domain, (semaphorin) 6A	2.79
ILMN_28730	similar to protein kinase, DNA-activated, catalytic polypeptide; protein kinase, DNA-activated, catalytic polypeptide	2.39
ILMN_17250	solute carrier family 1 (glial high affinity glutamate transporter), member 3	2.53
ILMN_11307	sortilin 1	-2.03
ILMN_5286	tetratricopeptide repeat domain 3; tetratricopeptide repeat domain 3-like	3.54
ILMN_16372	tumor necrosis factor, alpha-induced protein 2	2.18
ILMN_9324	twist homolog 1 (Drosophila)	2.20
ILMN_25778	versican	2.82
ILMN_21728	wingless-type MMTV integration site family, member 3A	-2.25

Regulation of Transcription		
**Genbank Accession no.**	**Protein Name**	**Ave Fold Δ**
ILMN_17477	AT rich interactive domain 5B (MRF1-like)	2.48
ILMN_2259	CCR4-NOT transcription complex, subunit 1	-2.02
ILMN_20291	CREB/ATF bZIP transcription factor	2.25
ILMN_1724	D site of albumin promoter (albumin D-box) binding protein	3.16

Regulation of Transcription (*Continued*)		
**Genbank Accession no.**	**Protein Name**	**Ave Fold Δ**
ILMN_19730	E2F transcription factor 2	-2.34
ILMN_24725	E2F transcription factor 8	-2.02
ILMN_13615	E74-like factor 3 (ets domain transcription factor, epithelial-specific)	2.80
ILMN_8384	PR domain containing 1, with ZNF domain	-2.43
ILMN_4347	SERTA domain containing 2	2.96
ILMN_10011	SMAD family member 7	-2.23
ILMN_17156	TH1-like (Drosophila)	-2.01
ILMN_24821	activating signal cointegrator 1 complex subunit 1	-2.36
ILMN_6018	activating signal cointegrator 1 complex subunit 3	2.05
ILMN_1659	apolipoprotein B mRNA editing enzyme, catalytic polypeptide-like 3F	2.18
ILMN_15884	apolipoprotein B mRNA editing enzyme, catalytic polypeptide-like 3G	2.10
ILMN_7180	basic leucine zipper transcription factor, ATF-like 3	-2.26
ILMN_2684, ILMN_30355	cyclin-dependent kinase inhibitor 2A (melanoma, p16, inhibits CDK4)	-2.52
ILMN_6524	cysteine-serine-rich nuclear protein 1	-2.22
ILMN_10721	early growth response 2	-2.67
ILMN_25740	enhancer of zeste homolog 2 (Drosophila)	-2.37
ILMN_17131	epiregulin	-2.63
ILMN_16913	ets homologous factor	5.02
ILMN_23624	forkhead box C1	-3.12
ILMN_138374	forkhead box D1	-2.29
ILMN_10159	forkhead box N2	2.23
ILMN_9965	forkhead box Q1	2.26
ILMN_18641	hairy/enhancer-of-split related with YRPW motif 1	-2.36
ILMN_15541	homeobox C4	2.55
ILMN_4115	interferon regulatory factor 7	3.95
ILMN_4335	mediator complex subunit 22	2.00
ILMN_137891	msh homeobox 1	-3.03
ILMN_10519	nuclear receptor coactivator 3	2.17
ILMN_7200	nuclear receptor subfamily 2, group F, member 1	-6.66
ILMN_28405	nuclear receptor subfamily 4, group A, member 2	-2.58
ILMN_804	nuclear receptor subfamily 4, group A, member 3	-2.33
ILMN_30051	nucleosomal binding protein 1	2.50
ILMN_17074	ovo-like 2 (Drosophila)	-2.45
ILMN_10778	peroxisomal proliferator-activated receptor A interacting complex 285	2.23
ILMN_7980	peroxisome proliferator-activated receptor gamma	-2.01
ILMN_27588	pre-B-cell leukemia homeobox 1	2.33
ILMN_10669	similar to transducin-like enhancer of split 1 (E(sp1) homolog, Drosophila); transducin-like enhancer of split 1 (E(sp1) homolog, Drosophila)	2.33
ILMN_9797	splicing factor, arginine/serine-rich 8 (suppressor-of-white-apricot homolog, Drosophila)	-2.03
ILMN_18876	transcription factor AP-2 alpha (activating enhancer binding protein 2 alpha)	4.11
ILMN_24479	tripartite motif-containing 24	2.29

Regulation of Transcription (*Continued*)		
**Genbank Accession no.**	**Protein Name**	**Ave Fold Δ**
ILMN_9324	twist homolog 1 (Drosophila)	2.20
ILMN_5708	v-ets erythroblastosis virus E26 oncogene homolog 1 (avian)	2.80
ILMN_13830	vacuolar protein sorting 36 homolog (S. cerevisiae)	2.86
ILMN_9797	splicing factor, arginine/serine-rich 8 (suppressor-of-white-apricot homolog, Drosophila)	-2.03
ILMN_18876	transcription factor AP-2 alpha (activating enhancer binding protein 2 alpha)	4.11
ILMN_24479	tripartite motif-containing 24	2.29
ILMN_9324	twist homolog 1 (Drosophila)	2.20
ILMN_5708	v-ets erythroblastosis virus E26 oncogene homolog 1 (avian)	2.80
ILMN_13830	vacuolar protein sorting 36 homolog (S. cerevisiae)	2.86
ILMN_10297	zinc finger protein 20	2.47
ILMN_12055	zinc finger protein 266	2.70
ILMN_17231	zinc finger protein 395	3.12
ILMN_2849	zinc finger protein 419	2.12
ILMN_13082	zinc finger protein 426	-2.02
ILMN_22117	zinc finger protein 45	2.50
ILMN_24422	zinc finger protein 702 pseudogene	2.62
ILMN_15160	zinc finger protein 816A	2.50
ILMN_23108	zinc finger protein 83	2.91
ILMN_6363	zinc finger protein 84	2.82
ILMN_13294	zinc finger protein 91	2.18

Regulation of Cell Communication		
**Genbank Accession no.**	**Protein Name**	**Ave Fold Δ**
ILMN_12195	6-phosphofructo-2-kinase/fructose-2,6-biphosphatase 2	2.32
ILMN_8233	ArfGAP with SH3 domain, ankyrin repeat and PH domain 2	2.07
ILMN_20228	DNA-damage-inducible transcript 4-like	-2.24
ILMN_4727	FYVE, RhoGEF and PH domain containing 6	2.07
ILMN_5586	KIT ligand	-2.57
ILMN_21302	NPC-A-7; bone marrow stromal cell antigen 2	2.93
ILMN_13405	RAP1 GTPase activating protein	2.69
ILMN_23476	S100 calcium binding protein P	-3.69
ILMN_10011	SMAD family member 7	-2.23
ILMN_20610	TBC1 domain family, member 15	2.47
ILMN_3919	TBC1 domain family, member 19	-2.70
ILMN_1046	TIMP metallopeptidase inhibitor 2	4.25
ILMN_23732	actin filament associated protein 1-like 2	2.07
ILMN_23272	amyloid beta (A4) precursor protein	2.19
ILMN_27187	bone morphogenetic protein 4	-3.20
ILMN_7507, ILMN_28609	brain-derived neurotrophic factor	7.39
ILMN_1624	caveolin 1, caveolae protein, 22kDa	3.05
ILMN_5108	caveolin 2	2.86
ILMN_2442	chromosome 5 open reading frame 13	2.21
ILMN_20121	cyclin-dependent kinase inhibitor 2B (p15, inhibits CDK4)	-3.06
ILMN_16536	cytohesin 4	2.15
ILMN_27871	dedicator of cytokinesis 7	-22.54
ILMN_5440	dual specificity phosphatase 6	-5.84
ILMN_10721	early growth response 2	-2.67
ILMN_25740	enhancer of zeste homolog 2 (Drosophila)	-2.37

Regulation of Cell Communication (*Continued*)		
**Genbank Accession no.**	**Protein Name**	**Ave Fold Δ**
ILMN_15615	epidermal growth factor receptor (erythroblastic leukemia viral (v-erb-b) oncogene homolog, avian)	2.23
ILMN_17131	epiregulin	-2.63
ILMN_18897	fibroblast growth factor 19	-2.23
ILMN_1771	fibroblast growth factor 9 (glia-activating factor)	-3.80
ILMN_13466	glutamate receptor, ionotropic, N-methyl D-aspartate 2C	-2.38
ILMN_22730, ILMN_11197	glutaredoxin 2	-2.11
ILMN_22105	guanine nucleotide binding protein (G protein), gamma 7	2.02
ILMN_18432	insulin receptor substrate 1	-2.23
ILMN_25529	insulin-like growth factor binding protein 3	3.05
ILMN_12662	integrin, alpha 2 (CD49B, alpha 2 subunit of VLA-2 receptor)	-4.25
ILMN_11560	kallikrein-related peptidase 6	7.71
ILMN_13886	membrane associated guanylate kinase, WW and PDZ domain containing 1; CNKSR family member 3	-2.08
ILMN_28251, ILMN_9993	microtubule-associated protein 1B	3.00
ILMN_3874	mitogen-activated protein kinase kinase kinase 5	-2.03
ILMN_10246	mitogen-activated protein kinase kinase kinase kinase 5	2.37
ILMN_26971	neuronal guanine nucleotide exchange factor	2.17
ILMN_10519	nuclear receptor coactivator 3	2.17
ILMN_17074	ovo-like 2 (Drosophila)	-2.45
ILMN_3538	phosphatidic acid phosphatase type 2B	2.00
ILMN_1289	plasminogen activator, tissue	-6.39
ILMN_28109	pleckstrin homology domain containing, family G (with RhoGef domain) member 3	2.03
ILMN_15492	proprotein convertase subtilisin/kexin type 9	2.02
ILMN_29986	prostaglandin-endoperoxide synthase 2 (prostaglandin G/H synthase and cyclooxygenase)	-2.21
ILMN_18896	protein tyrosine phosphatase, receptor type, F	2.51
ILMN_11345	signal-induced proliferation-associated 1 like 2	-5.50
ILMN_10669	similar to transducin-like enhancer of split 1 (E(sp1) homolog, Drosophila); transducin-like enhancer of split 1 (E(sp1) homolog, Drosophila)	2.33
ILMN_17250	solute carrier family 1 (glial high affinity glutamate transporter), member 3	2.53
ILMN_5176	solute carrier family 44, member 2	2.28
ILMN_4882	thrombospondin 1	2.33
ILMN_7641	transglutaminase 2 (C polypeptide, protein-glutamine-gamma-glutamyltransferase)	3.48
ILMN_24479	tripartite motif-containing 24	2.29
ILMN_2873	ubiquitin specific peptidase 46	-2.17

Regulation of Cell Proliferation		
**Genbank Accession no.**	**Protein Name**	**Ave Fold Δ**
ILMN_24441	CD33 molecule	-7.92
ILMN_22634	CD47 molecule	2.13
ILMN_1724	D site of albumin promoter (albumin D-box) binding protein	3.16
ILMN_5586	KIT ligand	-2.57
ILMN_1046	TIMP metallopeptidase inhibitor 2	4.25
ILMN_27187	bone morphogenetic protein 4	-3.20
ILMN_7507, ILMN_28609	brain-derived neurotrophic factor	7.39
ILMN_1624	caveolin 1, caveolae protein, 22kDa	3.05
ILMN_5108	caveolin 2	2.86
ILMN_3062	cyclin-dependent kinase 6	-2.36
ILMN_2684, ILMN_30355	cyclin-dependent kinase inhibitor 2A (melanoma, p16, inhibits CDK4)	-2.52
ILMN_20121	cyclin-dependent kinase inhibitor 2B (p15, inhibits CDK4)	-3.06
ILMN_29728	cysteine-rich protein 2	-3.67
ILMN_26123	ecotropic viral integration site 1	2.82
ILMN_15615	epidermal growth factor receptor (erythroblastic leukemia viral (v-erb-b) oncogene homolog, avian)	2.23
ILMN_17131	epiregulin	-2.63
ILMN_12939	fatty acid binding protein 6, ileal	5.07
ILMN_11546	ferritin, heavy polypeptide 1; ferritin, heavy polypeptide-like 16; similar to ferritin, heavy polypeptide 1; ferritin, heavy polypeptide-like 3 pseudogene	2.05
ILMN_1771	fibroblast growth factor 9 (glia-activating factor)	-3.80
ILMN_9313	fibroblast growth factor binding protein 1	2.65
ILMN_13020	heparin-binding EGF-like growth factor	-2.78
ILMN_18432	insulin receptor substrate 1	-2.23
ILMN_14503	insulin receptor substrate 2	2.33
ILMN_25529	insulin-like growth factor binding protein 3	3.05
ILMN_5216	insulin-like growth factor binding protein 6	3.19
ILMN_12662	integrin, alpha 2 (CD49B, alpha 2 subunit of VLA-2 receptor)	-4.25
ILMN_2247	interleukin 8	-4.35
ILMN_7322	laminin, beta 1	-4.95
ILMN_9188	matrix metallopeptidase 7 (matrilysin, uterine)	2.08
ILMN_137891	msh homeobox 1	-3.03
ILMN_17483	neuropilin 1	-2.77
ILMN_17074	ovo-like 2 (Drosophila)	-2.45
ILMN_19894	parathyroid hormone 1 receptor	2.06
ILMN_7980	peroxisome proliferator-activated receptor gamma	-2.01
ILMN_24167	plasminogen activator, urokinase	2.11
ILMN_27588	pre-B-cell leukemia homeobox 1	2.33
ILMN_2778	prostaglandin E synthase	2.62
ILMN_3001	prostaglandin-endoperoxide synthase 1 (prostaglandin G/H synthase and cyclooxygenase)	2.75
ILMN_29986	prostaglandin-endoperoxide synthase 2 (prostaglandin G/H synthase and cyclooxygenase)	-2.21
ILMN_18896	protein tyrosine phosphatase, receptor type, F	2.51
ILMN_17676	tensin 3	2.33
ILMN_11441	tetraspanin 31	2.03

Regulation of Cell Proliferation (*Continued*)		
**Genbank Accession no.**	**Protein Name**	**Ave Fold Δ**
ILMN_4882	thrombospondin 1	2.33
ILMN_7641	transglutaminase 2 (C polypeptide, protein-glutamine-gamma-glutamyltransferase)	3.48
ILMN_24479	tripartite motif-containing 24	2.29
ILMN_25430	tryptophanyl-tRNA synthetase	-4.74
ILMN_5708	v-ets erythroblastosis virus E26 oncogene homolog 1 (avian)	2.80

Lipid Metabolic Process		
**Genbank Accession no.**	**Protein Name**	**Ave Fold Δ**
ILMN_14756	1-acylglycerol-3-phosphate O-acyltransferase 4 (lysophosphatidic acid acyltransferase, delta)	2.28
ILMN_9737	1-acylglycerol-3-phosphate O-acyltransferase 5 (lysophosphatidic acid acyltransferase, epsilon)	-2.19
ILMN_21049	ATP-binding cassette, sub-family A (ABC1), member 1	5.28
ILMN_22988	DDHD domain containing 2	2.19
ILMN_30044	acetyl-Coenzyme A acyltransferase 1	2.45
ILMN_6029, ILMN_6741	acyl-CoA synthetase long-chain family member 5	-2.64
ILMN_25111	aldehyde dehydrogenase 1 family, member A3	3.82
ILMN_26797	aldehyde dehydrogenase 3 family, member B1	2.58
ILMN_1624	caveolin 1, caveolae protein, 22kDa	3.05
ILMN_24076	choline phosphotransferase 1	-2.11
ILMN_21296	cytochrome P450, family 24, subfamily A, polypeptide 1	-17.96
ILMN_20221	cytochrome P450, family 4, subfamily F, polypeptide 3	2.17
ILMN_12939	fatty acid binding protein 6, ileal	5.07
ILMN_12496	insulin induced gene 1	4.12
ILMN_28744	lipin 1	3.48
ILMN_2030	low density lipoprotein receptor-related protein 8, apolipoprotein e receptor	2.98
ILMN_17785	membrane bound O-acyltransferase domain containing 2	2.45
ILMN_25425	nephronophthisis 3 (adolescent); acyl-Coenzyme A dehydrogenase family, member 11	-2.13
ILMN_7980	peroxisome proliferator-activated receptor gamma	-2.01
ILMN_3538	phosphatidic acid phosphatase type 2B	2.00
ILMN_5923	phosphoinositide-3-kinase, regulatory subunit 1 (alpha)	2.62
ILMN_27588	pre-B-cell leukemia homeobox 1	2.33
ILMN_15492	proprotein convertase subtilisin/kexin type 9	2.02
ILMN_2778	prostaglandin E synthase	2.62
ILMN_3001	prostaglandin-endoperoxide synthase 1 (prostaglandin G/H synthase and cyclooxygenase)	2.75
ILMN_29986	prostaglandin-endoperoxide synthase 2 (prostaglandin G/H synthase and cyclooxygenase)	-2.21
ILMN_671	protein kinase, AMP-activated, gamma 2 non-catalytic subunit	2.02
ILMN_11289	retinol binding protein 1, cellular	-2.21
ILMN_521	squalene epoxidase	2.11

Cell Motility		
Genbank Accession no.	Protein Name	Ave Fold Δ
ILMN_17477	AT rich interactive domain 5B (MRF1-like)	2.48
ILMN_5586	KIT ligand	-2.57
ILMN_23476	S100 calcium binding protein P	-3.69
ILMN_15630	SATB homeobox 2	-2.25
ILMN_5108	caveolin 2	2.86
ILMN_7282	collagen triple helix repeat containing 1	-5.09
ILMN_139036	collagen, type V, alpha 1	-2.35
ILMN_3374	connective tissue growth factor	3.35
ILMN_13178	dynein, axonemal, heavy chain 2	3.69
ILMN_18897	fibroblast growth factor 19	-2.23
ILMN_23624	forkhead box C1	-3.12
ILMN_13020	heparin-binding EGF-like growth factor	-2.78
ILMN_2247	interleukin 8	-4.35
ILMN_2030	low density lipoprotein receptor-related protein 8, apolipoprotein e receptor	2.98
ILMN_17483	neuropilin 1	-2.77
ILMN_7200	nuclear receptor subfamily 2, group F, member 1	-6.66
ILMN_28405	nuclear receptor subfamily 4, group A, member 2	-2.58
ILMN_17074	ovo-like 2 (Drosophila)	-2.45
ILMN_3538	phosphatidic acid phosphatase type 2B	2.00
ILMN_1289	plasminogen activator, tissue	-6.39
ILMN_24167	plasminogen activator, urokinase	2.11
ILMN_12926	poly (ADP-ribose) polymerase family, member 9	2.25
ILMN_29382	sema domain, immunoglobulin domain (Ig), short basic domain, secreted, (semaphorin) 3C	2.03
ILMN_28730	similar to protein kinase, DNA-activated, catalytic polypeptide; protein kinase, DNA-activated, catalytic polypeptide	2.39
ILMN_17676	tensin 3	2.33
ILMN_4882	thrombospondin 1	2.33
ILMN_9324	twist homolog 1 (Drosophila)	2.20
ILMN_5708	v-ets erythroblastosis virus E26 oncogene homolog 1 (avian)	2.80
ILMN_25778	versican	2.82

Regulation of Cell Death		
Genbank Accession no.	Protein Name	Ave Fold Δ
ILMN_25111	aldehyde dehydrogenase 1 family, member A3	3.82
ILMN_23272	amyloid beta (A4) precursor protein	2.19
ILMN_20440	angiopoietin-like 4	-2.05
ILMN_3897	baculoviral IAP repeat-containing 3	2.61
ILMN_27187	bone morphogenetic protein 4	-3.20
ILMN_7507, ILMN_28609	brain-derived neurotrophic factor	7.39
ILMN_7434	caspase 4, apoptosis-related cysteine peptidase	2.41
ILMN_2684, ILMN_30355	cyclin-dependent kinase inhibitor 2A (melanoma, p16, inhibits CDK4)	-2.52
ILMN_15615	epidermal growth factor receptor (erythroblastic leukemia viral (v-erb-b) oncogene homolog, avian)	2.23
ILMN_23624	forkhead box C1	-3.12

Regulation of Cell Death (*Continued*)		
**Genbank Accession no.**	**Protein Name**	**Ave Fold Δ**
ILMN_6829	inhibitor of DNA binding 3, dominant negative helix-loop-helix protein	-3.22
ILMN_25529	insulin-like growth factor binding protein 3	3.05
ILMN_30275	interferon induced with helicase C domain 1	2.02
ILMN_3874	mitogen-activated protein kinase kinase kinase 5	-2.03
ILMN_137891	msh homeobox 1	-3.03
ILMN_25768	mutS homolog 6 (E. coli)	2.08
ILMN_26971	neuronal guanine nucleotide exchange factor	2.17
ILMN_28405	nuclear receptor subfamily 4, group A, member 2	-2.58
ILMN_15492	proprotein convertase subtilisin/kexin type 9	2.02
ILMN_29986	prostaglandin-endoperoxide synthase 2 (prostaglandin G/H synthase and cyclooxygenase)	-2.21
ILMN_18896	protein tyrosine phosphatase, receptor type, F	2.51
ILMN_26935	serine/threonine kinase 3 (STE20 homolog, yeast)	2.11
ILMN_28730	similar to protein kinase, DNA-activated, catalytic polypeptide; protein kinase, DNA-activated, catalytic polypeptide	2.39
ILMN_11307	sortilin 1	-2.03
ILMN_4882	thrombospondin 1	2.33
ILMN_7641	transglutaminase 2 (C polypeptide, protein-glutamine-gamma-glutamyltransferase)	3.48
ILMN_5708	v-ets erythroblastosis virus E26 oncogene homolog 1 (avian)	2.80

Cell Cycle Process		
**Genbank Accession no.**	**Protein Name**	**Ave Fold Δ**
ILMN_17286	LFNG O-fucosylpeptide 3-beta-N-acetylglucosaminyltransferase	2.58
ILMN_19136	RAN, member RAS oncogene family	-2.69
ILMN_23272	amyloid beta (A4) precursor protein	2.19
ILMN_3062	cyclin-dependent kinase 6	-2.36
ILMN_2684, ILMN_30355	cyclin-dependent kinase inhibitor 2A (melanoma, p16, inhibits CDK4)	-2.52
ILMN_20121	cyclin-dependent kinase inhibitor 2B (p15, inhibits CDK4)	-3.06
ILMN_15615	epidermal growth factor receptor (erythroblastic leukemia viral (v-erb-b) oncogene homolog, avian)	2.23
ILMN_17131	epiregulin	-2.63
ILMN_2247	interleukin 8	-4.35
ILMN_3286	microtubule-actin crosslinking factor 1	-2.12
ILMN_25768	mutS homolog 6 (E. coli)	2.08
ILMN_19658	ras homolog gene family, member U	-3.21
ILMN_7290	sestrin 2	-2.07
ILMN_4882	thrombospondin 1	2.33

Regulation of Cell Adhesion		
**Genbank Accession no.**	**Protein Name**	**Ave Fold Δ**
ILMN_22634	CD47 molecule	2.13
ILMN_9982	FXYD domain containing ion transport regulator 5	2.63
ILMN_9074	Rho GDP dissociation inhibitor (GDI) beta	5.15
ILMN_10011	SMAD family member 7	-2.23
ILMN_3062	cyclin-dependent kinase 6	-2.36
ILMN_2684, ILMN_30355	cyclin-dependent kinase inhibitor 2A (melanoma, p16, inhibits CDK4)	-2.52
ILMN_20248	dipeptidyl-peptidase 4	2.94
ILMN_12662	integrin, alpha 2 (CD49B, alpha 2 subunit of VLA-2 receptor)	-4.25
ILMN_2247	interleukin 8	-4.35
ILMN_4021	laminin, alpha 4	-2.48
ILMN_5923	phosphoinositide-3-kinase, regulatory subunit 1 (alpha)	2.62
ILMN_4882	thrombospondin 1	2.33
ILMN_7641	transglutaminase 2 (C polypeptide, protein-glutamine-gamma-glutamyltransferase)	3.48

Regulation of Gland Development		
**Genbank Accession no.**	**Protein Name**	**Ave Fold Δ**
ILMN_13615	E74-like factor 3 (ets domain transcription factor, epithelial-specific)	2.80
ILMN_25111	aldehyde dehydrogenase 1 family, member A3	3.82
ILMN_27187	bone morphogenetic protein 4	-3.20
ILMN_1624	caveolin 1, caveolae protein, 22kDa	3.05
ILMN_5108	caveolin 2	2.86
ILMN_15615	epidermal growth factor receptor (erythroblastic leukemia viral (v-erb-b) oncogene homolog, avian)	2.23
ILMN_23624	forkhead box C1	-3.12
ILMN_18432	insulin receptor substrate 1	-2.23
ILMN_14503	insulin receptor substrate 2	2.33
ILMN_12662	integrin, alpha 2 (CD49B, alpha 2 subunit of VLA-2 receptor)	-4.25
ILMN_27588	pre-B-cell leukemia homeobox 1	2.33
ILMN_21728	wingless-type MMTV integration site family, member 3A	-2.25

Response to Hypoxia		
**Genbank Accession no.**	**Protein Name**	**Ave Fold Δ**
ILMN_25542, ILMN_10855	ATPase, Na+/K+ transporting, beta 1 polypeptide	3.25
ILMN_20440	angiopoietin-like 4	-2.05
ILMN_1624	caveolin 1, caveolae protein, 22kDa	3.05
ILMN_20248	dipeptidyl-peptidase 4	2.94
ILMN_12662	integrin, alpha 2 (CD49B, alpha 2 subunit of VLA-2 receptor)	-4.25
ILMN_28405	nuclear receptor subfamily 4, group A, member 2	-2.58
ILMN_1289	plasminogen activator, tissue	-6.39
ILMN_24167	plasminogen activator, urokinase	2.11
ILMN_4882	thrombospondin 1	2.33

Regulation of Cytoskeleton Organization		
**Genbank Accession no.**	**Protein Name**	**Ave Fold Δ**
ILMN_6603	LIM domain and actin binding 1	2.18
ILMN_1624	caveolin 1, caveolae protein, 22kDa	3.05
ILMN_5108	caveolin 2	2.86
ILMN_3286	microtubule-actin crosslinking factor 1	-2.12
ILMN_28251, ILMN_9993	microtubule-associated protein 1B	3.00
ILMN_12193	thymosin beta 15a; thymosin beta 15B	2.04
**Positive regulation of Immune System Process**		
**Genbank Accession no.**	**Protein Name**	**Ave Fold Δ**
ILMN_22634	CD47 molecule	2.13
ILMN_17131	epiregulin	-2.63
ILMN_12662	integrin, alpha 2 (CD49B, alpha 2 subunit of VLA-2 receptor)	-4.25
ILMN_29874	killer cell lectin-like receptor subfamily K, member 1	-2.02
ILMN_4882	thrombospondin 1	2.33
**Regulation of Angiogenesis**		
**Genbank Accession no.**	**Protein Name**	**Ave Fold Δ**
ILMN_18802	angiopoietin 2	-40.43
ILMN_20440	angiopoietin-like 4	-2.05
ILMN_4882	thrombospondin 1	2.33
ILMN_25430	tryptophanyl-tRNA synthetase	-4.74
**Regulation of Exocytosis**		
**Genbank Accession no.**	**Protein Name**	**Ave Fold Δ**
ILMN_29025	RAB3B, member RAS oncogene family	-2.52
ILMN_14093	synaptotagmin I	-2.40
ILMN_29216	transient receptor potential cation channel, subfamily V, member 6	-3.17
